# Religiosity, Mental Health and Substance Use among Black and Hispanic Adults during the First Six Months of the COVID-19 Pandemic in New York City

**DOI:** 10.3390/ijerph20095632

**Published:** 2023-04-25

**Authors:** Connie Svob, Susan X. Lin, Keely Cheslack-Postava, Michaeline Bresnahan, Renee D. Goodwin, Norbert Skokauskas, George J. Musa, Sidney H. Hankerson, Diane R. Dreher, Megan Ryan, Yi-Ju Hsu, Anna-Lena Jonsson-Cohen, Christina W. Hoven

**Affiliations:** 1 Division of Child and Adolescent Psychiatry, Department of Psychiatry, Columbia University-New York State Psychiatric Institute, New York, NY 10032, USA; 2 Department of Epidemiology, Mailman School of Public Health, Columbia University, New York, NY 10032, USA; 3 Center for Family and Community Medicine, Vagelos College of Physicians and Surgeons, Columbia University, New York, NY 10032, USA; 4 Department of Epidemiology and Biostatistics, Graduate School of Public Health & Health Policy, City University of New York, New York, NY 10027, USA; 5 Center for Child and Youth Mental Health and Child Protection, IPH, Norwegian University of Science and Technology, 7030 Trondheim, Norway; 6 Department of Psychiatry, Institute for Health Equity Research, Icahn School of Medicine at Mount Sinai, New York, NY 10029, USA; 7 Department of Chaplaincy Services, Northwell Health, Lenox Hill Hospital, New York, NY 10075, USA

**Keywords:** religion/spirituality, COVID-19 pandemic, substance use, health disparities

## Abstract

The purpose of this study was to investigate the association between personal religiosity, mental health, and substance use outcomes among Black and Hispanic adults during the first six months of the COVID-19 outbreak in New York City (NYC). Phone interviews were conducted with 441 adults to obtain information on all variables. Participants self-reported race/ethnicity as Black/African American (*n* = 108) or Hispanic (*n* = 333). Logistic regression were used to examine associations between religiosity, mental health, and substance use. There was a significant inverse association of religiosity and substance use. Religious people had a lower prevalence of drinking alcohol (49.0%) compared to non-religious people (67.1%). Religious people also had substantially lower prevalence of cannabis or other drug use (9.1%) in comparison to non-religious people (31%). After adjusting for age, sex, race/ethnicity, and household income, the association of religiosity with alcohol use and with cannabis/other drug use remained statistically significant. Despite restricted access to in-person religious activities and congregational supports, the findings suggest that religiosity itself may be helpful from a public health perspective, independent of serving as a conduit for other social services.

## 1. Introduction

From its start in 2020, the coronavirus (COVID-19) pandemic disproportionately impacted historically marginalized groups, particularly in the United States [1]. Black and Hispanic Americans, for example, experienced substantially higher rates of infection, hospitalization, and death from COVID-19 compared to non-Hispanic White Americans [2]. Moreover, inequities in socioeconomic characteristics (e.g., employment status and health insurance) across racial lines were exacerbated [3].

Turning to religion in times of crisis remains a fundamental part of the way people make sense of how sickness, death, and suffering fit into a larger scheme of life. Religiosity can strengthen social connectedness and contribute to overall health and well-being, including in times of collective crisis. Religion—a particular system of faith and worship of a personal God or gods—historically played a prominent role in the lives of Americans, and especially so for Black and Hispanic communities [4,5,6]. Religiosity often involves belief in a higher power and the importance of religion, as well as religious devotion and practices (e.g., religious service attendance, prayer, and reading religious texts). Within the first few months of the COVID-19 pandemic, the vast majority of Americans (86%), including nearly a quarter of people who claimed no religious affiliation at all, adopted prayer as a response to the COVID-19 outbreak [7]. Religiosity was generally associated with better mental health outcomes [8] and with religious service attendance constituting the greatest predictor of better mental health [9,10]. It is unclear, however, what the impact of religiosity—particularly, in the absence of in-person religious services—meant for mental health outcomes.

Religious organizations often serve as sources of informal counseling, companionship, and support in times of distress, especially for minoritized, low resource communities [11]. The shelter-in-place requirements that mandated closings of houses of worship to prevent COVID-19 severely limited such access. These public health regulations were especially disruptive in Black and Hispanic communities, where religious congregations are often the only point of contact for adults seeking help for mental health and substance use challenges [12]. Given the potential racial/ethnic and economic disparity of COVID-19 outcomes, as well as limited in-person religious service attendance, evaluating the association between personal religiosity and mental health outcomes among Black and Hispanic Americans was considered critical. As such, the purpose of this study was to investigate associations between personal religiosity, substance use, and mental health outcomes among Black and Hispanic adults in the initial American COVID-19 epicenter, New York City, during the first six months of the pandemic.

## 2. Materials and Methods

### 2.1. Participants

The COVID-19 Check-In study, from which this study’s sample was drawn, was initiated in late March 2020 and included randomly selected participants (*n* = 1211) and controls from three ongoing longitudinal epidemiological studies in the NYC metropolitan area. Two of those cohorts (Stress & Well-Being and the WTC Family) comprised individuals who were exposed to the 9/11 attack and the third cohort (Stress & Justice) comprised individuals primarily from the South Bronx, the Bronx being one of the five boroughs of NYC. The Check-In study (51% White and 49% non-White adults) was designed to determine the health and well-being of on-going study participants during the pandemic. This study’s sample *n* = 441 (age ≥ 18 years, mean age 39.2 years ± 15.1) included individuals whose self-reported race/ethnicity was Black/African American (*n* = 108) or Hispanic (*n* = 333). Therefore, the racial/ethnic composition was 75.5% Hispanic and 24.5% Black/African Americans. Religious denominations included: Protestant (18.1%), Catholic (40.1%), Jewish (0.9%), Muslim (0.7%), Orthodox Christian: Greek, Russian, Coptic (1.1%), Jehovah’s Witness 1.6%), Mormon (Latter Day Saints) (0.9%), Other Religion (10.9%), No Religion (25.2%).

### 2.2. Procedures

Questionnaires were written in English and translated/back-translated into Spanish. Interviews were conducted in the language chosen by the participant (English 79.4% and Spanish 20.6%). All interviews were conducted over the phone by trained interviewers and entered directly into a Qualtrics password-protected survey. Participants were not compensated and participated voluntarily. All participants provided informed consent after receiving a complete description of the study. The COVID-19 Check-In study was approved by the [Blocked for Blind Review] Institutional Review Board.

### 2.3. Measures

Participants were asked to provide information on the following.

Age

What is your date of birth? (MM/DD/YYYY)

The response was treated as a continuous variable.

Gender

Are you female or male?

The response was treated as a dichotomous variable: Female/Male.

Race

Please tell me the name of the group that best describes you. Are you… American Indian, Asian, Hawaiian or Pacific Islander, Black (or African American), Black of Hispanic Origin, Eskimo (or Alaskan Native), White (or Caucasian), White of Hispanic Origin, Another group

Indicating more than one response was possible. Individuals who identified as either Black/African American (not Hispanic) or Hispanic were selected for inclusion in this study.

Socioeconomic Status

Please listen to these figures and tell me which best represents your own personal income before taxes for the past year. Please include only your own salaries, wages, social security, bonuses, welfare and any other income. (Include child support or alimony.)

Less than $5000, $5000–9999, $10,000–19,999, $20,000–34,999, $35,000–54,999, $55,000–74,999, $75,000–99,999, $100,000–199,999, $200,000 or more, Refuse to answer, don’t know

The variable for household income was grouped as follows: Low < $35,000, Middle $35,000–$99,999, High ≥ $100,000, or Don’t know/refused (16%).

Religious Denomination

How would you describe your religious group? Are you …

Protestant (e.g., Lutheran, Episcopal, Methodist, Presbyterian, Baptist, Pentecostal, Seventh Day Adventist, Quaker), Catholic, Jewish, Muslim, Buddhist, Orthodox Christian (Greek, Russian, Coptic), Hindu, Jehovah’s Witness, Mormon (Latter Day Saints), Sikh, Another Religion, No Religion.

Religiosity

How would you describe yourself? Are you …

Very religious, Somewhat religious, Not religious, Refused to answer, Do not know

Very religious and Somewhat religious were combined into a single category for analysis and compared with Not religious.

Depression

Symptoms within the past two weeks were assessed with the Patient Health Questionnaire (PHQ-8) [13]; which consists of 8 items scored by participants on a scale of 0–3 (not at all, several days, more than half the days, nearly every day). Scores were derived from summing the value of each symptom. Participants were considered positive for moderate to severe depression based on cut-off scores of 10 or higher.

Anxiety

Anxiety within the past two weeks was assessed with the General Anxiety Disorder (GAD-7) questionnaire [14]; scored on the same 0–3 scale (not at all, several days, more than half the days, nearly every day). Scores were derived from summing the value of each symptom. Participants were considered positive for moderate to severe anxiety based on cut-off scores of 10 or greater.

Substance Use

Alcohol: Do you drink alcohol (including wine, beer, hard liquor)? No, Yes, Refused to answer, Don’t know

Cannabis or other drugs: Do you use marijuana or other drugs? No, Yes, Refused to answer, Don’t know

## 3. Statistical Analysis

All analyses were conducted in SPSS (PASW Statistics 18). Descriptive statistics were used to characterize demographics. Chi-square tests were used to examine the association of religiosity with mental health outcomes. Logistic regression were used to examine these associations adjusted for age, sex, race/ethnicity, household income, and source study. The cut-off value of statistical significance is *p* < 0.05.

## 4. Results

### 4.1. Study Population Characteristics

Out of 441 study participants, 71.2% were female (*n* = 314). The majority of participants (64.9%) reported being either somewhat or very religious and 35.1% reported being not religious. Overall, 55.3% of the study sample reported drinking alcohol and 16.8% reported use of cannabis or other drugs during the first months of the pandemic. Slightly more than one-fifth (21.3%) had moderate–severe depression and 22.9% had moderate–severe anxiety (see Table 1 for characteristics of the study population).

### 4.2. Association of Religiosity with Mental Health and Substance Use Outcomes

#### 4.2.1. Religiosity and Mental Health Outcomes

Religiosity was not associated with prevalence of depression (20.6% in the non-religious group versus 21.7% in the somewhat/very religious group; *p* = 0.80) or anxiety (19.4% in the non-religious group versus 24.8% in the somewhat/very religious group; *p* = 0.19).

#### 4.2.2. Religiosity and Substance Use Outcomes

There was a significant cross-sectional association of religiosity and substance use. Religious people (i.e., those reporting to be somewhat or very religious) had a lower prevalence of drinking alcohol (49.0%) compared to non-religious people (67.1%). Religious people also had substantially lower prevalence of cannabis or other drug use (9.1%) compared to non-religious people (31.0%).

After adjusting for age, sex, race/ethnicity, household income, and source study, the association of religiosity with alcohol use and with cannabis/other drug use remained statistically significant. Compared to the non-religious, religious people had lower odds of alcohol use (adjusted OR: 0.62, 95% CI: 0.39–0.98) and cannabis use (adjusted OR: 0.35, 95% CI: 0.20–0.61) (see Table 2).

## 5. Discussion

During America’s first six months of the pandemic, with New York City comprising the nation’s initial COVID-19 epicenter, despite restricted access to in-person religious activities, religiosity among Black and Hispanic New Yorkers in this sample was associated with lower prevalence of alcohol, cannabis, or other drug use. There was no significant association observed between religiosity and depression or anxiety. These findings suggest that religiosity may not have been associated with a reduction in depressive and anxious symptoms in the initial shock of facing a global pandemic, but it may have been protective against substance use. The findings also suggest that disparities in substance use in socially marginalized groups, at least during the first six months of the pandemic, may have been aided by religious support among those who consider themselves to be religious. 

The findings complement findings of other studies. For example, according to a recent systematic review on spirituality and health, religious service attendance was shown to be associated with less smoking, as well as lower levels of alcohol use and drug use [15]. It was also associated with better mental health and better quality of life, including fewer depressive symptoms and lower levels of suicidal ideations and behaviors. However, religious service attendance in the first six months of the COVID-19 pandemic in New York City was restricted and it is unclear how this might have impacted the mental health and substance use of otherwise religious people. Despite limited access to in-person religious services, some studies from the time of the COVID-19 pandemic reported that religiosity/spirituality was, nonetheless, a commonly used coping strategy. For example, in New York City, specifically, 46% of healthcare workers reported using religion/spirituality and meditation to cope with stresses associated with the COVID-19 outbreak [16]. 

In general, findings on the association between religiosity and mental health outcomes during the COVID-19 pandemic have been mixed. Many have reported religiosity/spirituality to be associated with decreased symptoms of mental disorders and increased well-being. For example, higher levels of religious coping in Muslims, compared to their Christian counterparts, revealed an inverse relationship between religious coping and current symptom levels of depression in the Muslim cohort [17]. Religious coping was further shown to moderate the relationship between hope and well-being in people observing stringent lockdown regulations [18]. Similar findings were also observed beyond New York and the USA. For example, in a sample of Palestinian adults, an inverse correlation was observed between positive religious coping and perceived stress, as well as a negative correlation between positive religious coping and depressive symptoms [19]. 

On the other hand, and consistent with our findings, several studies failed to find an association between religiosity and mental health outcomes. For example, no association was observed in the relationship between mental health and various religious beliefs as measured by the Duke University Religious Index (DUREL) [20]. Lack of an association was also observed in another study that investigated the relationship between religiosity and COVID-related anxiety in a sample of health care workers in Portugal [21].

The fact that there was limited access to houses of worship and in-person religious services at the height of the COVID-19 outbreak in New York City, and at the time of this study, may account for the lack of an association between reported religiosity and symptoms of anxiety and depression. It is possible that restricting in-person religious service attendance decreased the likelihood that religiosity would be associated with lower rates of anxiety and depression. Moreover, limiting access to in-person religious services may have reduced people’s tendency to adopt a religious identity and to seek solace in religious beliefs and practices. 

It should also be noted that symptoms of anxiety and depression tend to focus on affective states, rather than behaviors. Religiosity may not have shielded individuals from the negative affect (i.e., symptoms of depression and anxiety) spurred by the COVID-19 pandemic, but it may have, nonetheless, been associated with protective effects on behavioral outcomes, as observed in alcohol and drug consumption. Rather than using substances to cope with the pandemic’s stressors, religious individuals may have turned to faith to help regulate potentially destructive coping strategies, despite limited access to in-person congregational supports and services. 

The paucity and, hence, diversity of studies on religion and health during the COVID-19 pandemic may also account for the mixed associations reported in the literature as several variables remain inconsistent across studies and complicate comparability across studies, including geographic locations, religious groups sampled, religious/spiritual measures, mental health and substance use outcomes, and the timeframe of the study within the (to date, 3-year) COVID-19 pandemic. Despite these limitations, the present study contributes to the complex literature uniquely by adding a voice for historically minoritized groups—Black and Hispanic Americans.

There are several implications of the study’s findings—not only in North America, but globally, as well. The findings suggest that guided public health outreach by churches and religious groups may be beneficial to those who are religious; these may include social outreach to isolated members, access to resources for addiction and substance use, and information sharing about vaccinations and other supportive services. The findings also support the potential value of chaplaincy outreach as the role of chaplaincy is to support religiosity through spiritual awareness, solace, and a connection to the sacred at times when access to in-person services may not be possible—amid a pandemic or otherwise. 

The study’s findings, although limited by their cross-sectional design and single item measure of self-reported religiosity, contribute to research that investigates religiosity as a potentially protective and supportive mechanism in times of crisis. They hint at the possibility that religiosity might help reduce substance use and suggest that religiosity itself may be helpful from a public health perspective, independent of serving as a conduit for other social services. They also support the possibility that in-person religious services are important for mental health and limiting access to these services (including, in some cases, sacraments) may have repercussions of which we are little aware. It is a limitation that we did not assess the specific religious practices in which respondents engaged. Also, the study was cross-sectional in design, so no causal claims can be made. Further study is needed to assess how specific religious/spiritual factors relate to mental health and substance use in times of collective crisis. As such, in-depth, longitudinal examination of religiosity (e.g., prayer, access to online religious services) is warranted.

## 6. Conclusions

Historically marginalized groups in the US were shown to face widespread social and institutional disadvantages. For Black and Hispanic communities, the most religiously oriented groups, religiosity may serve as a resilient factor in the face of adversity. The COVID-19 pandemic provided a unique opportunity to assess the association between religious community support and mental health and substance use outcomes when access to in-person congregational supports were restricted. We found that individuals who identified as being religious maintained the positive effects often associated with religiosity—in this case, lower rates of substance use—as compared to those who did not identify as religious. This finding suggests that an internal religious orientation (e.g., faith, spirituality) may sustain some of the benefits of in-person religious supports when they cannot be accessed. This has implications beyond lockdown in a pandemic—it provides important insight for clinicians, counselors, chaplains, psychiatrists, social scientists, religious leaders, and policy makers. More research is merited.

## Figures and Tables

**Table 1:** Characteristics of the study population (*n* = 441).

	*n* = 441	%
Race/Ethnicity		
Black	108	24.5
Hispanic	333	75.5
Age (mean years/SD)	39.2/15.1	
18–39	208	47.2
40–65	224	50.8
65+	9	2.0
Sex		
Male	127	28.8
Female	314	71.2
Household Income		
Low	125	28.3
Middle	158	35.8
High	89	20.2
Unknown	69	15.6

**Table 2:** Association of religiosity with substance use and mental health outcomes.

	Prevalence of Drinking Alcohol	AOR * (95% CI)	*p*-Value
Not Religious	67.1	Referent	0.04
Somewhat/Very Religious	49.0	0.62 (0.39–0.98)	
	**Prevalence of Cannabis/Other Drug Use**		
Not Religious	31.0	Referent	<0.01
Somewhat/Very Religious	9.1	0.35 (0.20–0.61)	
	**Prevalence of Moderate/Severe Depression**		
Not Religious	20.6	Referent	0.54
Somewhat/Very Religious	21.7	1.17 (0.70–1.97)	
	**Prevalence of Moderate/Severe Anxiety**		
Not Religious	19.4	Referent	0.29
Somewhat/Very Religious	24.8	1.32 (0.79–2.23)	

* Adjusted Odds Ratio (AOR) for age, sex, race/ethnicity, household income, and source study.

## Data Availability

The data presented in this study are available on request from the corresponding author. The data are not publicly available due to privacy restrictions.
